# Ассоциация предоперационной терапии колекальциферолом и гипокальциемии после паратиреоидэктомии у больных с первичным гиперпаратиреозом

**DOI:** 10.14341/probl13324

**Published:** 2024-02-28

**Authors:** А. Р. Елфимова, А. К. Еремкина, О. Ю. Реброва, Е. В. Ковалева, Н. Г. Мокрышева

**Affiliations:** Национальный медицинский исследовательский центр эндокринологии; Национальный медицинский исследовательский центр эндокринологии; Национальный медицинский исследовательский центр эндокринологии; Национальный медицинский исследовательский центр эндокринологии; Национальный медицинский исследовательский центр эндокринологии

**Keywords:** первичный гиперпаратиреоз, гипокальциемия, колекальциферол, паратиреоидэктомия

## Abstract

**ОБОСНОВАНИЕ:**

ОБОСНОВАНИЕ. Первичный гиперпаратиреоз (ПГПТ) — распространенное эндокринное заболевание, возникающее в результате гиперсекреции паратиреоидного гормона (ПТГ) опухолью околощитовидных желез (ОЩЖ). Единственным радикальным методом лечения ПГПТ является паратиреоидэктомия (ПТЭ). Частота гипокальциемии после ПТЭ может достигать 46% случаев. При этом значимое снижение уровня кальция в сыворотке крови ассоциировано как с локальными, так и с генерализованными судорогами тонико-клонического типа, жизнеугрожающими нарушениями сердечного ритма. Сопутствующий дефицит витамина D может усугубить тяжесть течения ПГПТ и способствовать развитию в раннем послеоперационном периоде «синдрома голодных костей», характеризующегося тяжелой и стойкой гипокальциемией вследствие резкого снижения уровня ПТГ и усиленного «оттока» кальция в обедненную костную ткань.

**ЦЕЛЬ:**

ЦЕЛЬ. Оценить наличие ассоциации и показатели силы связи предоперационной терапии колекальциферолом с развитием гипокальциемии через 1–3 дня после ПТЭ у больных с ПГПТ.

**МАТЕРИАЛЫ И МЕТОДЫ:**

МАТЕРИАЛЫ И МЕТОДЫ. Обследование проводилось в ФГБУ «НМИЦ эндокринологии» Минздрава России в 1993–2010 или 2017–2020 гг. Критериями включения было наличие у пациентов ПГПТ и показаний к ПТЭ, сывороточный уровень 25-гидроксивитамина D (25(OH)D)<20 нг/мл и уровень общего кальция в сыворотке <3 ммоль/л. Критерием исключения являлась терапия препаратами, влияющими на кальциево-фосфорный обмен, — цинакальцетом, деносумабом или бисфосфонатами (как в виде монотерапии, так и в составе комбинированной терапии).

**РЕЗУЛЬТАТЫ:**

РЕЗУЛЬТАТЫ. В исследование было включено 117 пациентов, из них 110 (94%) женщин и 7 (6%) мужчин. Медиана и квартили возраста составили 58 [49; 65] лет. 21 (18%) пациент принимал колекальциферол от 2 недель до 2 месяцев до ПТЭ в дозе, соответствующей восполнению дефицита витамина D; 96 (82%) пациентов не принимали колекальциферол. Группы пациентов, принимающих и не принимающих колекальциферол, были сопоставимы по антропометрическим (пол, возраст на момент операции), предоперационным клиническим (снижение МПК) и лабораторным показателям (ПТГ, общий кальций, фосфор, ЩФ, ОК, СТХ-1, 25(OH)D). Послеоперационная гипокальциемия статистически значимо реже наблюдалась у пациентов, принимающих колекальциферол (10% против 63%, p<0,001, ТКФ2). Прием колекальциферола отрицательно ассоциирует с развитием гипокальциемии (ОР=0,15, 95% ДИ (0,03; 0,51)).

**ЗАКЛЮЧЕНИЕ:**

ЗАКЛЮЧЕНИЕ. Прием колекальциферола от 2 недель до 2 месяцев перед ПТЭ снижает риск послеоперационной гипокальциемии у пациентов с ПГПТ в 2–33 раза.

## Abstract (en)

**BACKGROUND:**

BACKGROUND: Primary hyperparathyroidism (PHPT) is a endocrine disorder characterized by excessive secretion of parathyroid hormone (PTH) from parathyroid gland tumors. Parathyroidectomy (PTE) is the main treatment for PHPT, but it can lead to hypocalcemia in up to 46% of cases. Hypocalcemia is associated with seizures and life-threatening cardiac arrhythmias, and vitamin D deficiency can exacerbate PHPT severity and contribute to «hungry bones syndrome,» resulting in severe and persistent postoperative hypocalcemia.

**AIM:**

AIM: To evaluate the association and determine the strength of the relationship between preoperative cholecalciferol therapy and the occurrence of hypocalcemia within 1–3 days after PTE in patients with PHPT.

**MATERIALS AND METHODS:**

MATERIALS AND METHODS: The study was conducted at the Endocrinology Research Centre, during the periods of 1993–2010 and 2017–2020. The inclusion criteria consisted of patients diagnosed with PHPT who required PTE, had a serum 25-hydroxyvitamin D (25(OH)D) level below 20 ng/mL, and a serum total calcium level below 3 mmol/L. The exclusion criterion was the use of medications that affect calcium-phosphorus metabolism, including cinacalcet, denosumab, or bisphosphonates, either as monotherapy or as part of combination therapy.

**RESULTS:**

RESULTS: There were 117 patients, including 110 (94%) females and 7 (6%) males. The median age and interquartile range were 58 [49; 65] years. Among the participants, 21 (18%) received cholecalciferol supplementation for a duration of 2 weeks to 2 months prior to PTE, aiming to address vitamin D deficiency. The remaining 96 (82%) participants did not receive ­cholecalciferol supplementation. Both groups, i.e., participants receiving cholecalciferol and those who did not, were similar in terms of anthropometric factors (sex and age at the time of surgery), preoperative clinical characteristics (BMD decrease), and laboratory parameters (PTH, total calcium, phosphorus, ALP, OC, CTX-1, and 25(OH)D levels). The occurrence of postoperative hypocalcemia was significantly lower in participants who received cholecalciferol supplementation (10% vs. 63%, p<0,001, FET2). Cholecalciferol intake showed a negative association with hypocalcemia development (RR=0,15, 95% CI (0,03; 0,51)).

**CONCLUSION:**

CONCLUSION: Preoperative cholecalciferol supplementation for 2 weeks to 2 months before PTE reduces the risk of postoperative hypocalcemia in patients with PHPT by 2–33 times.

## ОБОСНОВАНИЕ

Первичный гиперпаратиреоз (ПГПТ) представляет собой распространенное эндокринное заболевание, характеризующееся избыточной секрецией паратиреоидного гормона (ПТГ) новообразованиями околощитовидных желез (ОЩЖ) [1]. Избыточная секреция ПТГ приводит к гиперкальциемии за счет увеличения реабсорбции кальция в почечных канальцах, стимуляции остеокласт-опосредованной резорбции кости и увеличения синтеза кальцитриола (1,25(OH)2D3), что, в свою очередь, способствует усилению всасывания кальция и фосфатов в кишечнике. Данные патогенетические механизмы лежат в основе поражения костной и почечной системы, проявляющиеся снижением минеральной плотности костей (МПК), низкоэнергетическими переломами (НЭП), развитием нефрокальциноза/нефролитиаза, а также снижением фильтрационной и концентрационной функции почек.

Единственным радикальным методом лечения ПГПТ является паратиреоидэктомия (ПТЭ), в то время как консервативная терапия остается актуальной для предоперационной коррекции гиперкальциемии и уменьшения потери МПК [2][3]. Пациенты с ПГПТ могут получать лечение на предоперационном этапе в виде моно- или комбинированной терапии: нативная форма витамина D (колекальциферол), антирезорбтивная терапия и кальцимиметики.

Следует отметить, что при ПГПТ нередко наблюдается недостаточность или дефицит витамина D. Предполагается, что дефицит витамина D может стать начальным звеном в патогенезе ПГПТ, способствуя гиперплазии ОЩЖ с последующим формированием автономной секрецией ПТГ и трансформацией в аденому [4]. Существуют данные, указывающие на то, что сопутствующий дефицит витамина D связан с более тяжелым течением ПГПТ, более высоким повышением уровня ПТГ и как следствие более тяжелой гиперкальциемией [5]. Кроме того, дефицит витамина D при ПГПТ ассоциирован с тяжелыми костными нарушениями, более низкими показателями МПК, а также более высоким риском развития «синдрома голодных костей» после ПТЭ [10–13]. Назначение колекальциферола с целью восполнения дефицита витамина D при ПГПТ может устранить вторичное повышение уровня ПТГ в крови. Однако пациентам с уровнем общего кальция в сыворотке крови 3,0 ммоль/л и выше не рекомендуется назначать данную терапию в связи с риском прогрессирования гиперкальциемии и угрозы гиперкальциемического криза.

Учитывая тот факт, что назначение терапии колекальциферолом проводится далеко не всем пациентам в активной стадии ПГПТ, актуальным остается вопрос о взаимосвязи между наличием дефицита витамина D и развитием послеоперационной гипокальциемии. ПТЭ может осложняться послеоперационной гипокальциемией в 46% случаев [6] и проявляться различными симптомами, такими как судороги, миалгия, нарушения сердечного ритма и т.д. Безусловно, послеоперационная гипокальциемия представляет собой многофакторное состояние, и помимо дефицита витамина D, свой вклад вносят такие важные факторы, как «синдром голодных костей», повреждение здоровых ОЩЖ и др. [7] Синдром «голодных костей» обычно развивается в ранний послеоперационный период у пациентов с тяжелыми костными проявлениями ПГПТ. Это связано с выраженным снижением уровня кальция в крови, которое происходит из-за устранения стимулирующего эффекта гиперпродукции ПТГ на остеокластическую резорбцию костей, что приводит к активному переходу кальция из крови в формирующуюся костную ткань. По данным большинства исследований, «синдром голодных костей» ассоциирован с дооперационным наличием субпериостальных эрозий, литическими поражениями костей, «бурыми» опухолями, множественными переломами. Фиброзно-кистозный остеит может определяться у 47–100% пациентов с данным синдромом. Другими прогностически неблагоприятными факторами считаются вес и размер аденомы ОЩЖ. Существующие исследования, направленные на оценку связи между риском развития «синдрома голодных костей» и различными лабораторными показателями (в частности уровнями ПТГ, кальция, щелочной фосфатазы, остеокальцина, С-концевого телопептида коллагена 1 типа, витамина D), дают противоречивые результаты. Эффективные методы профилактики этого состояния до сих пор недостаточно изучены [6].

В последнее время появляется все больше данных о необходимости предоперационной коррекции дефицита витамина D [8][9]. Насыщение витамином D на дооперационном этапе может предотвратить развитие транзиторной послеоперационной гипокальциемии, однако результаты исследований на эту тему противоречивы. В ряде случаев ПТЭ может привести к развитию стойкого гипопаратиреоза, хотя его частота не превышает 3% случаев [10][11].

Считается, что уровень ПТГ является не только критерием эффективности, проведенной ПТЭ по поводу ПГПТ, но и надежным маркером послеоперационного гипопаратиреоза [12][13]. Однако развитие гипокальциемии не всегда сопоставимо с уровнем ПТГ. Пациент может быть выписан из стационара до того, как уровень кальция в сыворотке начнет снижаться (как правило резкое снижение отмечается через 24–72 часа после хирургического вмешательства). В связи с чем практикующему врачу, с одной стороны, важно знать факторы риска развития послеоперационной гипокальциеимии, а с другой стороны, уметь предупреждать развитие данного патологического состояния. Кроме того, несмотря на актуальность данной проблемы, исследования, посвященные эффекту приема колекальциферола до проведения ПТЭ, ограничены. Имеются данные о том, что дефицит витамина D является независимым фактором риска транзиторной гипокальциемии после тотальной тиреоидэктомии [8][9]. Однако в настоящее время исследований, направленных на изучение влияния предоперационной терапии ПГПТ на возникновение послеоперационной гипокальциемии, недостаточно.

## ЦЕЛЬ ИССЛЕДОВАНИЯ

Цель настоящего исследования заключалась в оценке наличия ассоциации и показателей силы связи между предоперационной терапией колекальциферолом и гипокальциемией в раннем послеоперационном периоде (1–3 сутки после ПТЭ) у пациентов с ПГПТ.

## МАТЕРИАЛЫ И МЕТОДЫ

## Место и время проведения исследования

Место проведения. Источник случаев: стационарные отделения ФГБУ «НМИЦ эндокринологии» Минздрава России.

Время исследования. Периоды включения пациентов: 1993–2010 и 2018–2020 гг.

## Изучаемые популяции (одна или несколько)

Целевая популяция определялась критериями включения и исключения.

Критерии включения: установленный диагноз спорадического ПГПТ (код МКБ-10 Е21.0), проведение радикальной ПТЭ по поводу ПГПТ, предоперационная сывороточная концентрация 25(OH) витамина D (25(OH)D) ниже 20 нг/мл (дефицит витамина D) [14], предоперационная сывороточная концентрация общего кальция ниже 3 ммоль/л (в соответствии с действующими клиническими рекомендациями, колекальциферол небезопасен для пациентов с гиперкальциемией >3 ммоль/л) [2][15], достижение нормализации сывороточного ПТГ и кальция на 1–3 сутки после операции.

Критерии исключения: отсутствие данных о концентрации кальция и ПТГ в сыворотке, измеренных на 1–3 день после ПТЭ, прием антирезорбтивной терапии или кальцимиметиков перед ПТЭ.

Способ формирования выборки из изучаемой популяции (или нескольких выборок из нескольких изучаемых популяций)

Способ формирования выборки — сплошной.

## Дизайн исследования

Проведено проспективное одноцентровое исследование. График исследования представлен на рисунке 1.

**Figure:**
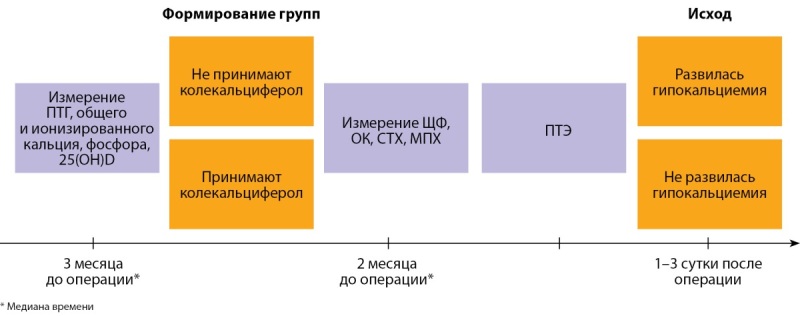
Рисунок 1. График исследования ассоциации приема колекальциферола с развитием гипокальциемии в раннем послеоперационном периоде (сокращения: 25(OH)D — 25(OH) витамин D, ЩФ — щелочная фосфатаза, ОК — остеокальцин, СТХ — С-концевой телопептид коллагена I типа, МПК — минеральная плотность кости, ПТЭ — паратиреоидэктомия).

В рамках исследования регистрировались следующие показатели: ПТГ, общий кальций, фосфор (измеряли за 5 дней — 4 года до операции и до терапии колекальциферолом при ее наличии); щелочная фосфатаза (ЩФ), остеокальцин (ОК), С-концевой телопептид коллагена 1 типа (СТХ-1), 25(OH)D и минеральную плотность костей (МПК) (измеряли за 4–365 дней до операции). Регистрируемым исходом являлось развитие гипокальциемии на 1–3 сутки после ПТЭ. Гипокальциемия определялась как концентрация общего кальция в сыворотке ниже нижнего референсного диапазона — <2,15 ммоль/л при нормальной концентрации альбумина в плазме [16].

## Методы

Диагноз ПГПТ устанавливался на основании стойкого повышения уровня ПТГ или высоконормального уровня ПТГ в сочетании с дважды подтвержденным повышенным уровнем кальция крови, или сочетания нормокальциемии с повышенным уровнем ПТГ после исключения вторичных причин гиперпаратиреоза [2].

Измерение МПК осуществлялось с помощью рентгеновской двухэнергетической денситометрии. Оценивались следующие отделы: шейка бедренной кости (femur neck), бедренная кость (total hip), треть лучевой кости (radius 33%), лучевая кость (radius total), поясничный отдел позвоночника (L1-L4). Снижение МПК определялось по T- и Z-критериям, которые представляют собой стандартное отклонение (SD) от среднего значения пика костной массы нормы в соответствующих половозрастных группах. T-критерий (T-кр.) для женщин в менопаузе и мужчин 50 лет и старше; Z-критерий (Z-кр.) — для женщин до менопаузы, мужчин в возрасте моложе 50 лет [21–24].

Оценка радикальности ПТЭ была проведена в соответствии с клиническим рекомендациями 2020 г.: уровень интраоперационного ПТГ должен нормализоваться или снизиться на 50% и более от исходного значения через 15 минут после удаления опухоли ОЩЖ [2]. ПТЭ у всех пациентов была выполнена в отделе хирургии ФГБУ «НМИЦ эндокринологии» Минздрава России.

## Статистический анализ

Статистический анализ проведен в программном пакете Statistica 13 (Tibco, США) и в среде R3.6.3. Описательная статистика количественных данных представлена медианами, первым и третьим квартилями в формате Me [Q1; Q3], качественных — в виде абсолютных и относительных частот. Критерий Манна-Уитни (U-тест) использовали для сравнения количественных признаков двух независимых групп. Для сравнения групп по частотам бинарных признаков использовали точный двусторонний критерий Фишера (ТКФ2).

Ассоциация между фактом приема колекальциферола и развитием послеоперационной гипокальциемии оценивалась показателем относительного риска (ОР) и его 95% доверительным интервалом (ДИ).

Критический уровень статистической значимости при проверке статистических гипотез принят равным 0,05. При множественных сравнениях применялась поправка Бонферрони путем коррекции критического уровня значимости.

## Этическая экспертиза

Протокол исследования одобрен локальным этическим комитетом ФГБУ «НМИЦ эндокринологии» Минздрава России (протокол №1 от 17.01.2018).

Все пациенты ознакомились с информацией и подписали информированное согласие на участие в процедурах, использование их биологического материала, обработку персональных данных до того, как приняли участие в исследовании.

## РЕЗУЛЬТАТЫ

Всего в исследование включено 117 пациентов, из них 7 (6%) мужчин и 110 (94%) женщин. Медиана возраста составила 58 [ 49; 65] лет. Из 117 пациентов 21 (18%) принимал колекальциферол минимум в течение двух недель перед ПТЭ согласно потребности (в зависимости от текущего уровня 25(OH)D), 96 (82%) пациентов не принимали терапию по поводу ПГПТ.

В период с 2018 по 2020 гг. было прооперировано 85 (73%) пациентов, в то время как в период с 1993 по 2010 гг. — 32 (27%) пациента. Статистически значимо более высокий уровень ЩФ был обнаружен у пациентов, прооперированных с 1993 по 2010 гг., по сравнению с пациентами, прооперированными с 2018 по 2020 гг. (табл. 1). Эти различия, вероятно, связаны с отсутствием скрининга кальция и более поздним диагнозом симптомного ПГПТ в предыдущие десятилетия.

**Table:** Таблица 1. Сравнительный анализ пациентов, пролеченных в 1993–2010 и 2018–2022 гг. (n=117) Поправка Бонферрони P0=0,05/11=0,005.¹ТКФ2.² U-тест.

N	Пациенты, пролеченные в 1993–2010 гг. (n=32)	Пациенты, пролеченные в 2018–2020 гг. (n=85)	p
N	Me [ Q1; Q3] / n (%)	N	Me [ Q1; Q3] / n (%)
Пол, муж.	32	3 (9%)	85	4 (5%)	0,390¹
Возраст, годы	32	56 [ 42; 63]	85	59 [ 51; 65]	0,151²
МПК	SD (T-кр.)≥-1,0/SD (Z-кр.)≥-2,0	32	6 (19%)	79	22 (28%)	0,618¹
-1,0>SD (T-кр.)≥-2,5	4 (13%)	9 (11%)
SD (T-кр.)<-2,5/SD (Z-кр.)<-2,0	17 (53%)	32 (41%)
SD (T-кр.)<-2,5+НЭП/SD (Z-кр.)<-2,0+НЭП	5 (16%)	16 (21%)
ПТГ, пг/мл	32	171,6 [ 115,4; 292,2]	85	141,6 [ 11,0; 186,6]	0,010²
Кальций общий, ммоль/л	32	2,75 [ 2,64; 2,85]	85	2,75 [ 2,65; 2,84]	0,699²
Фосфор, ммоль/л	32	0,82 [ 0,77; 0,98]	76	0,89 [ 0,78; 0,98]	0,497²
ЩФ, Ед/л	31	265 [ 207; 315]	76	92 [ 72; 108]	<0,001²
ОК, нг/мл	32	47,2 [ 31,3; 73,5]	65	47,4 [ 34,5; 74,4]	0,997²
СТХ, нг/мл	32	0,97 [ 0,69; 1,16]	73	0,87 [ 0,63; 1,36]	0,817²
25(OH)D, нг/мл	32	11,8 [ 9,3; 14,5]	85	13,6 [ 10,2; 17,4]	0,079²
Гипокальциемия	32	19 (59%)	85	43 (51%)	0,415¹

Группы пациентов, получавшие и не получавшие колекальциферол, с учетом поправки Бонферрони (Р0=0,005) статистически значимо не различались по демографическим, клиническим и лабораторным показателям (табл. 2), что свидетельствует о сопоставимости групп.

**Table:** Таблица 2. Сравнительный анализ групп пациентов, принимавших и не принимавших колекальциферол (n=117) Поправка Бонферрони P0=0,05/11=0,005.¹ Двусторонний ТКФ2.² U-тест.

N	Пациенты, получавшие колекальциферол(n=21)	Пациенты, не получавшие медикаментозную терапию (n=96)	p
N	Me [ Q1; Q3] / n (%)	N	Me [ Q1; Q3] / n (%)
Пол, муж.	21	0 (0%)	96	7 (7%)	0,349¹
Возраст, годы	21	62 [ 50; 65]	96	57 [ 47; 65]	0,288²
МПК	SD (T-кр.)≥-1,0/SD (Z-кр.)≥-2,0	21	4 (19%)	90	24 (27%)	0,133¹
-1,0>SD (T-кр.)≥-2,5	2 (10%)	11 (12%)
SD (T-кр.)<-2,5/SD (Z-кр.)<-2,0	7 (33%)	42 (47%)
SD (T-кр.)<-2,5+НЭП/SD (Z-кр.)<-2,0+НЭП	8 (38%)	13 (14%)
ПТГ, пг/мл	21	123 [ 110; 161]	96	149 [ 117; 247]	0,031²
Кальций общий, ммоль/л	21	2,74 [ 2,61; 2,82]	96	2,75 [ 2,65; 2,85]	0,432²
Фосфор, ммоль/л	17	0,97 [ 0,85; 1,01]	91	0,87 [ 0,76; 0,96]	0,019²
ЩФ, Ед/л	19	98 [ 78; 109]	88	109 [ 82; 224]	0,083²
ОК, нг/мл	18	50,9 [ 37,9; 74,4]	79	47,3 [ 31,5; 74,3]	0,967²
СТХ, нг/мл	20	0,88 [ 0,58; 1,39]	85	0,93 [ 0,65; 1,29]	0,571²
25(OH)D, нг/мл	21	14,00 [ 10,50; 18,29]	96	12,44 [ 9,54; 16,05]	0,120²
Гипокальциемия	21	2 (10%)	96	60 (63%)	<0,001¹

При анализе ассоциаций предоперационного приема нативной формы витамина D и развития гипокальциемии в раннем послеоперационном периоде обнаружено, что частота гипокальциемии в группе колекальциферола примерно в 6 раз меньше (табл. 2), а ОР составляет 0,15, 95% ДИ (0,03; 0,51). Таким образом, прием колекальциферола снижает риск развития послеоперационной гипокальциемии в 2–33 раза (рис. 2).

**Figure:**
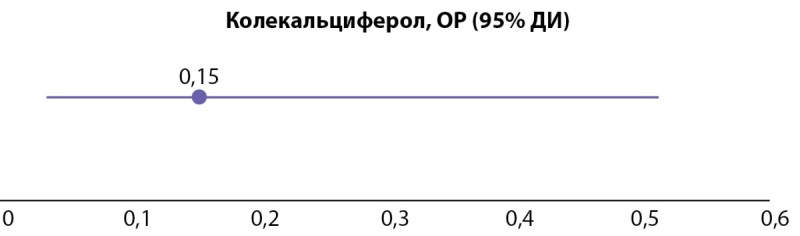
Рисунок 2. ОР (95% ДИ) развития гипокальциемии на 1–3 сутки после ПТЭ в зависимости от предоперационного приема колекальциферола.

## ОБСУЖДЕНИЕ

Результаты данного исследования согласуются с ранее проведенными, в которых изучался статус витамина D у пациентов с развившейся послеоперационной гипокальциемией. Прием колекальциферола перед проведением хирургического лечения на области шеи способствует восполнению дефицита или недостаточности 25(OH)D и, таким образом, может предотвратить развитие транзиторной гипокальциемии. Кроме того, прием витамина D перед операцией способствует улучшению состояния костной ткани, что снижает риск возникновения «синдрома голодных костей» и связанной с ним гипокальциемии [21].

Большинство работ, посвященных риску развития послеоперационной гипокальциемии и ее связи с дефицитом витамина D, проводились в популяции пациентов с заболеваниями щитовидной железы. Метаанализ Edafe и соавт. показал, что уровень предоперационного 25(OH)D является предиктором гипокальциемии после тиреоидэктомии [8]. Erbil и соавт. провели сравнительный анализ предоперационного уровня 25(OH)D в сыворотке крови в двух группах пациентов с болезнью Грейвса: с гипокальциемией и с нормокальциемией, перенесших тотальную тиреоидэктомию. Предоперационный уровень 25(OH)D в сыворотке был статистически значимо ниже в группе пациентов с гипокальциемией (9,7 против 13,2 нг/мл, р=0,010, U-тест) [9].

Исследования, посвященные проблеме развития гипокальциемии после ПТЭ, лимитированы. У наших пациентов, принимавших колекальциферол, статистически значимо реже наблюдалась послеоперационная гипокальциемия по сравнению с пациентами, не принимавшими данный препарат. Результаты трех различных исследований сходны с нашими данными и подтверждают протективную роль нормальной концентрации 25(OH)D [7][21][22]. Исследование, проведенное Acharya и соавт., показало, что насыщение витамином D является значимым фактором, влияющим на симптомы гипокальциемии. Полученные результаты свидетельствуют о наличии статистически значимой ассоциации между насыщением витамином D и развитием гипокальциемии (ОШ=4,9 [ 95% ДИ: 1,8–13,7]) [7]. В исследовании Unsal и соавт. было обнаружено, что развитие транзиторной гипокальциемии статистически значимо чаще наблюдается у пациентов с предоперационным дефицитом и недостаточностью витамина D [22]. Это указывает на то, что дефицит витамина D является независимым фактором риска для развития транзиторной гипокальциемии после ПТЭ. Кроме того, в проспективном исследовании, проведенном Salman и соавт., была рассмотрена роль витамина D в профилактике «синдрома голодных костей» после успешной ПТЭ по поводу ПГПТ. Исходный уровень 25(OH)D в сыворотке пациентов с «синдромом голодных костей» был значительно ниже, чем у пациентов без данного синдрома (11,0 против 17,3 нг/мл) [21]. Эти результаты подтверждают значимость витамина D в поддержании нормального метаболизма костной ткани после ПТЭ. В то же время Press и соавт. не выявили различий в частоте симптомов гипокальциемии и потребности в кальции после операции у пациентов с различным предоперационным уровнем витамина D. Анализ показал, что уровень витамина D у пациентов с очень низкими (<20 нг/мл), низкими (от 21 до 30 нг/мл), нормальными (>30 нг/мл) и низкими значениями с добавлением витамина D (исходно<25 нг/мл, после терапии колекальциферолом >40 нг/мл) не оказывал значимого влияния на наличие симптомов гипокальциемии и потребность в кальции после ПТЭ [23].

Несмотря на противоречивые результаты, большинство исследований свидетельствуют, что у пациентов с нормальным уровнем витамина D шансы развития гипокальциемии после ПТЭ ниже, что согласуется с данными нашей работы. Таким образом, мы считаем, что крайне важно проводить предоперационное насыщение колекальциферолом пациентам в активной стадии ПГПТ. В соответствии с действующими клиническими рекомендациями, восполнение сопутствующего дефицита (недостаточности) витамина D на дооперационном этапе показано пациентам с сывороточным кальцием не более 3 ммоль/л (<12 мг/дл). Эта рекомендация основана на имеющихся данных по безопасности приема колекальциферола именно среди лиц с мягкой гиперкальциемией. По результатам метаанализа 10 наблюдательных исследований (n=340), использование данного препарата в насыщающих дозировках (до 100 000 МЕ в неделю) на этапе предоперационной подготовки сопровождалось снижением уровня иПТГ, достоверным увеличением 25(ОН)D и сохранением исходных показателей сывороточного кальция и суточной кальциурии в большинстве случаев. В 2,2% случаев было зарегистрировано нарастание гиперкальциемии, повлекшее за собой отмену препаратов. В данный метаанализ вошли исследования с бессимптомным течением ПГПТ со средним уровнем гиперкальциемии 2,7–2,8 ммоль/л. Пациентам с ПГПТ и уровнем кальция >3 ммоль/л (>12 мг/дл) не рекомендуется восполнение сопутствующего дефицита (недостаточности) витамина D до проведения ПТЭ в виду отсутствия данных о безопасности назначения препарата. Тем не менее в случае наличия умеренной или выраженной гиперкальциемии в качестве первого терапевтического агента можно было бы порекомендовать назначить кальцийснижающую терапию (кальцимиметиками или антирезорбтивными препаратами в зависимости от показаний) и далее, по достижению кальциемии менее 3 ммоль/л, провести насыщение витамином D. Более того, пациенты со значимым повышением уровня кальция в сыворотке крови, как правило, характеризуются более выраженными изменениями со стороны костной ткани и более высокими значениями иПТГ. Рандомизированное двойное слепое исследование, посвященное оценке эффективности и безопасности назначения колекальциферола в дозе 2800 МЕ в сутки в течение 6 месяцев до и после операции, продемонстрировало значимое снижение уровня исходного иПТГ c достижением оптимальных значений 25(ОН)D, при этом показатели кальциемии и суточной кальциурии сохранялись без изменений. Также терапия колекальциферолом по сравнению с плацебо была ассоциирована с увеличением МПК в поясничном отделе позвоночника на 2,5% (p=0,01) и снижением маркера костной резорбции бета-кросслапса на 22% (р<0,005) [24]. Таким образом, приведенные исследования демонстрируют необходимость поддержания нормального уровня витамина D у пациентов с ПГПТ в предоперационном периоде с целью снижения риска гипокальциемии после ПТЭ.

## Ограничения исследования

В связи с тем, что исследуемые группы относятся к разным историческим промежуткам времени, в исследовании присутствует историческое смещение, в частности по уровню ЩФ. В группе, относящейся к более раннему периоду, уровень ЩФ был статистически значимо выше (р<0,001, U-тест).

## ЗАКЛЮЧЕНИЕ

Прием колекальциферола от 2 недель до 2 месяцев перед проведением ПТЭ снижает риск развития послеоперационной гипокальциемии у пациентов с ПГПТ в диапазоне от 2 до 33 раз.

## ДОПОЛНИТЕЛЬНАЯ ИНФОРМАЦИЯ

Источники финансирования. Статья опубликована в рамках выполнения государственного задания «Оптимизация российского электронного реестра пациентов с первичным гиперпаратиреозом» № НИОКТР 121030100032-7 при финансовой поддержке Министерства здравоохранения Российской Федерации.

Конфликт интересов. Авторы декларируют отсутствие явных и потенциальных конфликтов интересов, связанных с публикацией настоящей статьи.

Участие авторов. Елфимова А.Р. — получение и анализ данных, интерпретация результатов, создание иллюстративного материала, написание статьи; Еремкина А.К. — разработка концепции и дизайна исследования, интерпретация результатов, редактирование текста статьи; Реброва О.Ю. — разработка концепции и дизайна исследования, интерпретация результатов, редактирование текста статьи; Ковалева Е.В. — интерпретация результатов, редактирование текста статьи; Мокрышева Н.Г. — разработка концепции и дизайна исследования, внесение в рукопись существенных правок.

Все авторы одобрили финальную версию статьи перед публикацией, выразили согласие нести ответственность за все аспекты работы, подразумевающую надлежащее изучение и решение вопросов, связанных с точностью или добросовестностью любой части работы.
